# Integer programming as a powerful tool for polyclonal selection in ancient grapevine varieties

**DOI:** 10.1007/s00122-025-04885-0

**Published:** 2025-05-21

**Authors:** Sónia Surgy, Jorge Cadima, Elsa Gonçalves

**Affiliations:** 1 LEAF—Linking Landscape, Environment, Agriculture and Food—Research Center, Associated Laboratory TERRA, Instituto Superior de Agronomia, Universidade de Lisboa, Tapada da Ajuda, 1349-017 Lisbon, Portugal; 2 Instituto Superior de Agronomia, Universidade de Lisboa, Lisbon, Portugal

## Abstract

**Key message:**

Integer programming was used as a novel approach for grapevine selection. Several selection criteria were considered using real data to test the method, which was successfully applied to polyclonal selection.

**Abstract:**

Polyclonal selection (selecting a high-performing, balanced mixture of 7 to 20 clones) in ancient grapevine varieties is a selection method that is increasingly used in countries with ancient viticulture. However, to meet the needs of the vine and wine sector, polyclonal selection must take into account several target traits. Polyclonal selection is based on empirical best linear unbiased predictors of genotypic effects obtained by fitting appropriate linear mixed models. This work proposes a multicriteria method for polyclonal selection. A new approach based on integer programming is implemented to perform polyclonal selection considering multiple traits simultaneously. An algorithm that attempts to maximize the genetic gains of selection according to different selection criteria has been developed and tested on real data of important traits obtained in large field trials of four ancient grapevine varieties. Multiple selection criteria were used to perform polyclonal selection of groups of 7 to 20 clones of each variety based on multiple traits. The results showed that integer programming can be useful in polyclonal selection to obtain selected material with high genetic gains in the target traits, while avoiding losses in other equally important traits.

## Introduction

Grapevine domestication occurred thousands of years ago (McGovern et al. 2017) and is one of the most significant plant domestications in human history. Since then, wine has been present in cultural traditions such as religious rituals, and viticulture has great economic importance and a characteristic impact on the landscape.

Clonal selection is a method of improving ancient grapevine varieties and has been used since the 1950s, first to obtain virus-free clones and then to improve quality traits of the grapes, which initially were mainly yield and sugar content (Atak et al. 2014). However, clonal selection (the selection of individual clones) induces excessive genetic homogeneity in the vineyards leading to genetic erosion (Loureiro et al. 2011) and higher sensitivity to genotype-by-environment interaction (Gonçalves et al. 2016, 2019, 2020). Therefore, clonal selection should be based on evaluations from multienvironment field trials in the main growing regions of the traditional variety. As grapevine is a perennial crop, models for data analysis must combine the information across sites and across years within a site and account for the correlation induced by repeated measurements of the same plot along the years in the same site (Gonçalves et al. 2020).

However, the importance of conserving and evaluating intravarietal genetic variability has been recognized as the basis for genetic selection, and new approaches to grapevine selection have been developed. The Portuguese methodology for selection of ancient grapevines varieties (Martins and Gonçalves 2015; Gonçalves and Martins 2022) includes conservation and assessment of genetic variability within a variety and polyclonal selection. Polyclonal selection is therefore a methodology of selection applied to ancient grapevine varieties where it is possible for the exploration of intravarietal variability to respond to the objectives of the wine sector. Consequently, this method is employed in relation to traditional varieties cultivated in a known, adapted range of wine-producing regions and of major importance for the certification of wines from these regions.

Polyclonal selection can be defined as the selection of a group of different genotypes which, when considered as a whole, meet certain criteria for desirable quantitative traits. The focus, therefore, is on the collective behavior of the group rather than on the individual behavior of each clone within the group. In practice, the material cultivated in commercial vineyards is a balanced, indistinguishable mixture of clones (Teixeira et al. 2023). The use of polyclonal material helps to maintain a certain level of genetic variability in the vineyards, ensuring greater environmental stability. A more detailed analysis of the stabilizing effect of polyclonal material on environmental stability has been demonstrated in previous studies, specifically in Martins and Gonçalves (2015). The variation of this effect according to the size of the polyclonal group (groups of size 1, 2, up to 20) was studied in variety Arinto by comparing the behavior of these groups in 33 environments with the behavior of a group of 40 clones (representing the average environmental stability behavior of the variety). The results demonstrated that environmental stability increased with the number of clones in the group, and that the use of mixtures of at least seven clones ensured stability close to that of the average environmental stability behavior of the variety. The resolution of the International Organization of Vine and Wine (OIV) OIV-VITI 564B-2019 (OIV Resolution OIV-VITI 564B-2019, 2019) concerning the recovery and conservation of intravarietal diversity and polyclonal selection stipulates a minimum group size of 7 clones for polyclonal material to ensure minimum stability and a maximum of 20 clones to facilitate substantial genetic gains in selection relative to the mean of the variety. An important point to emphasize about this type of selection is that it makes it possible to predict the genetic gains of selection obtained for the variety. This is achieved by conducting a field trial, in which a random sample of the variety is evaluated and established with appropriate experimental designs (Gonçalves et al. 2010), and selection is made according to the principles of quantitative genetics (Gonçalves and Martins 2019). This methodology is described in detail in Resolution OIV-VITI 564B-2019 (OIV Resolution OIV-VITI 564B-2019, 2019) as well as in Gonçalves and Martins (2022). This is a selection method that is increasingly being used in countries with ancient viticulture.

Polyclonal selection should take into account several important traits simultaneously. Traits of economic and oenological interest such as yield and must quality traits are numerical variables. The variability of quantitative traits is assessed by their variance components (Falconer and Mackay 1996) which are obtained by fitting mixed models that consider genotype as a random effects factor. The parameters of these models are estimated by residual maximum likelihood (REML) method (Patterson & Thompson 1971). Genetic selection should be based on the empirical best linear unbiased predictors (EBLUPs) of genotypic effects (Henderson 1975; McCulloch et al. 2008). Several authors suggested selection indices as the most efficient method to select for several traits simultaneously (Cotterill and Dean 1990; Falconer and Mackay 1996). Smith (1936) developed the first selection index based on Fisher’s discriminant function. Since then, many indices have been proposed, but usually with two disadvantages. One is the risk of decreasing some trait values when improving others (Cotterill and Dean 1990), a risk that grows when there are negative correlations between traits. A second is the requirement of economic weights which are difficult to determine objectively (Cotterill and Jackson 1985; Falconer and Mackay 1996). The original selection index proposed by Smith (1936) was based on the best available genetic value of an individual (Smith 1936) which allows the ranking of all individuals and the identification and selection of those with the highest genetic values. The value of the index is also referred to as genetic worth by Cotterill and Dean (1990) since the coefficients of the traits are weighted by economic importance. Ideally, a selection index should be able to identify the best clones according to multiple traits. Nevertheless, selection indices were developed to identify superior individual genotypes rather than to maximize the gain for a collective behavior of a group of genotypes. As far as we are aware, no index has been developed with polyclonal selection in mind. But there are other options instead of defining an index. Using EBLUPs of genotypic effects, the identification of a group of superior clones can be seen as an optimization problem where the goal is to maximize the predicted genetic gain of the group as a whole, in accordance with the selection criteria adopted.

Multicriteria decision analysis (MCDA) techniques belong to a group of quantitative methods that use numerical information to evaluate several decision alternatives (Kaim et al. 2018). Malczewski (2006) considers that MCDA includes both multiattribute and multiobjective decision-making methods, denoted here as MADM and MODM, respectively. While MADM problems have a given small number of alternatives and can be solved through a complete search of all such alternatives, the best solution to a MODM problem can lie anywhere in the region of feasible solutions (Malczewski 2006). In the context of polyclonal selection, a group of clones that satisfy a given criterion is selected. This finding suggests that the utilization of MADM could be a viable option. However, the large number of alternatives makes it impossible to solve the problem by evaluation methods such as pairwise comparisons (Triantaphyllou 2000; Malczewski 2006). This kind of problem can be solved using mathematical optimization techniques (Uhde et al. 2015). Linear programming is a mathematical optimization technique that was developed in the 1950s and has had an extraordinary impact ever since (Hillier and Lieberman 2015). Linear programming allows the optimization (maximization or minimization) of a linear objective function, subject to several constraints, and has already been applied in genomic selection of cross breeding species (Moeinizade et al. 2019, 2020; Zhang and Wang 2022).

Integer programming (IP) is a special case of linear programming in which all decision variables are integers. It has not been previously used to perform polyclonal selection in ancient grapevine varieties. The aim of this work is to apply IP to polyclonal selection and to present an algorithm that attempts to maximize the genetic gains of selection according to different selection criteria. Some specific cases are tested with real data from selection field trials of ancient grapevine varieties.

## Materials and methods

### Plant material, evaluated traits, and experimental design

Four grapevine varieties were considered in this study: two red varieties, Grenache and Rufete, and two white varieties, Gouveio and Encruzado. In accordance with the sanitary certification legislation, a field trial was conducted for each variety, comprising a random sample of the variety. This sample was constituted by dozens of clones from mother plants that were prospected in old vineyards of the primary growing regions of the variety. This approach was adopted to ensure the presence of intravarietal variability. The number of clones (treatments) ranged from 126 to 242, depending on the variety studied. The field trials were established according to resolvable row–column designs, the specifications of which are described in Table 1.

**Table 1:** Description of the field trials for polyclonal selection in the four varieties used in this study: location, traits evaluated, experimental design, and years of evaluation

Variety (Location)	Trait	Resolvable row-column design					Years of evaluation
		N. of clones (treatments)	N. of resolvable replicates	N. of rows nested within the resolvable replicate	N. of columns nested within the resolvable replicate	N. of plants per experimental unit	
Encruzado (Alijó/Portugal)	Yield (YD, kg/plant)	180	4	10	18	3	2009;2012; 2014 to 2022
	Potential alcohol (PA, %v/v); Total Acidity (TA, g of tartaric acid/l); pH	180	3	10	18	3	2019 to 2021
Gouveio (Palmela/Portugal)	Yield (YD, kg/plant)	150	5	14	11	3	2020 to 2022
	Potential alcohol (PA, %v/v); Total Acidity (TA, g of tartaric acid/l); pH; Berry Weight (BW, g/berry)	150	3	14	11	3	2020 to 2022
Grenache (Palmela/Portugal)	Yield (YD, kg/plant)	126	6	6	21	3	2020 to 2022
	Potential alcohol (PA, %v/v); Total Acidity (TA, g of tartaric acid/l); pH; Berry Weight (BW, g/berry); Anthocyanin content (AC, mg/l); Total Phenols Index (TP)	126	3	6	21	3	2020
Rufete (Palmela/Portugal)	Yield (YD, kg/plant)	242	6	11	22	3	2020 to 2022
	Potential alcohol (PA, %v/v); Total Acidity (TA, g of tartaric acid/l); pH; Berry Weight (BW, g/berry); Anthocyanin content (AC, mg/l); Total Phenols Index (TP)	242	3	11	22	3	2020

Harvest yield (YD) was measured in all replicates of the field trial. On the day before harvest, 60 berries per experimental unit were collected. Due to the laborious and costly process of measuring berry traits, berry samples were only collected in three replicates of the field trial for the assessment of berry weight (BW) and must quality traits. After weighing, the berry samples were pressed. From the juice, pH was determined with a pH meter, ºBrix by refraction, and total acidity (TA) by titration (OIV, 2021). The potential alcohol (PA) was estimated at 17 ºBrix, corresponding to 10% potential alcohol by volume (Ribéreau-Gayon et al. 2006). For the red varieties, the skins and seeds were placed in flasks with a buffer solution of tartaric acid (pH 3.20) and ethanol 96%, which was macerated at 25 °C. After maceration, the extract was collected and centrifuged for 10 min at 3500 r.p.m. (Carbonneau and Champagnol 1993). This extract was used for the determination of anthocyanins (AC) and total phenolics (TP) by spectrophotometry at 520 nm and 280 nm, respectively (Ribéreau-Gayon and Stonestreet 1965). The results are expressed in kg/plant for YD, g/berry for BW, %vol for PA, g Tart. Ac./l for TA, and mg/g for AC.

Information on the traits evaluated and the years of evaluation is also given in Table 1. Given that the objective of the study was to perform polyclonal selection, the emphasis is on the aggregate behavior of the group of clones with respect to the traits over the course of several years. Consequently, the data analysis was based on the average values observed over several years for each trait per experimental unit (plot). The yearly value for each experimental unit is given by the average of the observed values for the three plants in each experimental unit. As the aim of this study was to test the application of a multicriteria polyclonal selection method and not to select a specific group for future use, data from berries of only one year were used to test the proposed method in the selection of the two red varieties.

### Statistical methods

#### The model

The number of replicates for yield was different from the number of replicates for quality traits of the must. Therefore, linear univariate mixed models were fitted for each trait considered (rather than a multitrait mixed model). The general univariate mixed model can be defined in matrix notation as

$${\varvec{Y}} = {\varvec{X\beta}} + {\varvec{Zu}} + {\varvec{e}}$$

${\varvec{Y}}_{{\left( {n \times 1} \right)}}$ is the random vector of observations (mean values for all years, in each experimental unit);

${\varvec{\beta}}_{{\left( {p \times 1} \right)}}$ is the vector of fixed effects (includes the overall mean and the main effects of the resolvable replicates);

${\varvec{X}}_{{\left( {n \times p} \right)}}$ is the design matrix of fixed effects;

${\varvec{u}}_{{\left( {q \times 1} \right)}}$ is the vector of random effects (includes clone effects, the effects of the rows within the resolvable replicate, the effects of the columns within the resolvable replicate) and $q = \sum\nolimits_{{i = 1}}^{3} {q_{i} } ,$ where $q_{i}$ is the number of levels of random effects factor $i$;

${\varvec{Z}}_{{\left( {n \times q} \right)}}$ is the design matrix of random effects;

${\varvec{e}}_{{\left( {n \times 1} \right)}}$ is the vector of random errors.

The vectors ${\varvec{u}}$ and ${\varvec{e}}$ are assumed mutually independent with multivariate normal distribution with vector of mean values ${\varvec{0}}_{{\left( {q \times 1} \right)}}$ and ${\varvec{0}}_{{\left( {n \times 1} \right)}}$ and covariance matrices ${\varvec{G}}_{{\left( {q \times q} \right)}}$ and ${\varvec{R}}_{{\left( {n \times n} \right)}}$, respectively. Consequently, the distribution of $\bf Y$ is multivariate normal with mean value ${\varvec{X\beta}}$ and covariance matrix ${\varvec{V}} = {\varvec{ZGZ}}^{T} + {\varvec{R}}$, where ${\varvec{Z}}^{T}$ is the transpose of ${\varvec{Z}}$. The vector ${\varvec{u}}$ takes the form ${\varvec{u}} = \left( {{\varvec{u}}_{g}^{T} ,{\varvec{u}}_{{row\left( {rep} \right)}}^{T} ,{\varvec{u}}_{{col\left( {rep} \right)}}^{T} } \right)^{T}$, where each sub-vector corresponds to a random effects factor, which are mutually independent and each with covariance matrix given by ${\varvec{G}}_{i} = \sigma_{i}^{2} {\varvec{I}}_{{q_{i} }} ,\forall i = 1, \ldots ,3$, where ${\varvec{I}}_{{q_{i} }}$ is the identity matrix of order $q_{i}$; Therefore, the covariance matrix of vector ${\varvec{u}}$ is defined as $\varvec{G} = \oplus _{{i = 1}}^{3} \varvec{G}_{i}$, where $\oplus$ denotes the direct sum of matrices.

The elements of the vector of random errors ${\varvec{e}}_{{\left( {n \times 1} \right)}}$ are assumed to be independent and identically distributed random variables, that is, ${\varvec{R}} = \sigma_{e}^{2} {\varvec{I}}_{n}$, where ${\varvec{I}}_{n}$ is the identity matrix of order $n$.

#### Quantification of intravarietal variability, prediction of genotypic effects, broad-sense heritability, and predicted genetic gain

Variance components were estimated by the restricted maximum likelihood method (REML) (Patterson and Thompson 1971). The genotypic variance component was tested using a residual maximum likelihood ratio test (REMLRT). For this hypothesis test $\left( {H_{0} :\sigma_{g}^{2} = 0 vs\,H_{1} :\sigma_{g}^{2} > 0} \right)$, the conservative solution was adopted (Stram and Lee 1994), that is, it was assumed that the asymptotic distribution of the test statistic is a Chi-square distribution with one degree of freedom.

For a more practical comparison of the quantification of intravarietal variability of traits within and between varieties, the coefficient of genotypic variation ($CV_{G}$) for each trait was calculated as the ratio between the estimate of the genotypic standard deviation and the overall mean of the trait.

For the model described above, the vector of the best linear unbiased predictors (EBLUPs) of the random effects is defined as (Henderson 1975; Searle et al. 1992)

$$\tilde{\textbf{u}}_{EBLUP} = \hat{{\varvec{G}}}{\varvec{Z}^{T}} \hat{{\varvec{V}}^{ - 1}} \left( {{\varvec{Y}} - {\varvec{X\hat{\beta }}_{EBLUE}} } \right),$$

where $\varvec{\hat{\beta }}_{EBLUE} = \left( {{\varvec{X}}^{T} \hat{{\varvec{V}}^{ - 1}} {\varvec{X}}} \right)^{ - 1} {\varvec{X}}^{T} \hat{{\varvec{V}}^{ - 1}} {\varvec{Y}}$ is the vector of the empirical best linear unbiased estimators (EBLUEs) of the fixed effects. For selection purposes, the focus is on the elements of the vector ${\varvec{\tilde u}}_{EBLUP}$ that relate to EBLUPs of genotypic effects for the trait being analyzed. EBLUPs of genotypic effects are deviations from the overall mean that can be explained by genetic causes and are therefore the basis for selection. The arithmetic mean of the EBLUPS of the genotypic effects of a superior group of selected clones is the predicted genetic gain of the selected group (i.e., of polyclonal selection), and this is intended to be as far from zero as possible.

Another important measure that was taken into consideration for the interpretation of the results obtained in the various field trials for the different traits was broad-sense heritability. According to Falconer and Mackay (1996), heritability is the squared correlation between the predicted and the true genetic effects (or, in other words, a measure of the proportion of phenotypic variation that is explained by genetic causes) and is therefore a measure related to the quality of the data collected in the field trial, directly related to the precision and the potential success of selection. In this work, a generalized measure of broad-sense heritability of a trait for a given grapevine selection field trial was defined as (Gonçalves et al. 2013):

$$H^{2} = \frac{{\mathop \sum \nolimits_{j = 1}^{{q_{1} }} \left( {1 - \frac{{PEV_{j} }}{{\sigma_{g}^{2} }}} \right)}}{t},$$

where $PEV_{j}$ is the prediction error variance of the genotypic effect for genotype j, $\left( {j = 1, \ldots ,q_{1} } \right), \sigma_{g}^{2}$ is the genotypic variance component, and $t$ is the rank of a matrix defined as $\varvec{I}_{{q_{1} }} - \varvec{G}_{g}^{ - 1} \varvec{C}_{gg}$ (where ${\varvec{I}}_{{q_{1} }}$ is the identity matrix of order $q_{1}$, ${\varvec{G}}_{g}$ is the covariance matrix of genotypic effects, and $\varvec{C}_{gg}$ is the prediction error variance matrix of genotypic effects).

In this work, only data with a broad-sense heritability greater than 0.30 were considered for polyclonal selection.

#### Selection using integer programming

In the present study, polyclonal selection was performed with consideration for multiple traits. In accordance with Resolution OIV-VITI 564B-2019 (OIV Resolution OIV-VITI 564B-2019, 2019), groups of 7 to 20 clones were selected through integer programming (IP) as the designated selection method. The objectives of selection should be defined according to the variety and its specific characteristics, as well as the needs of the wine sector. In this study, the following criteria were considered to test the application of IP: to increase the mean values of yield (YD), potential alcohol (PA), total acidity (TA), total phenols index (TP), and anthocyanin content (AC) and to decrease the mean values of berry weight (BW) and pH. Yield increases are of great importance for producers with a direct impact on their profits. However, an increase in yields should not be obtained by increasing berry weights, since it is generally accepted that smaller berries give rise to better quality wine (Chen et al. 2018). On the other hand, global warming is causing a lower acidity (and consequently a higher pH) which has a negative impact on wine parameters (Payan et al. 2023). However, the balance between total acidity and potential alcohol should be maintained. Furthermore, increasing anthocyanins content and total phenols relies on the strong relation that polyphenols have with wine quality and health-promoting properties (Gutiérrez-Escobar et al. 2021). Therefore, the values of BW and pH genotypic effects EBLUPs were multiplied by $-1$ prior to the application of the selection method, so that larger values are considered gains according to the proposed selection criteria.

In the context of selection, constraints are defined by the several traits. The general definition for the predicted genetic gain (R) for a trait in a specific polyclonal selection is given by the mean of the EBLUPs of the genotypic effects of this group of selected clones as a percentage of the overall mean of the corresponding trait. Using the EBLUP of genotypic effects divided by the trait mean is a way of maximizing the gain as a proportion of the mean. More importantly, EBLUPs have the same units of measurement as the traits with which they are associated. Dividing by the mean both creates dimensionless quantities and does away with the impact that the differing scales have on the magnitude of the EBLUPs. This procedure is essential to obtain meaningful solutions in the IP problem.

The general IP problem for each variety can be written as maximizing the *objective function*:

$$z = \mathop \sum \limits_{i = 1}^{n} \mathop \sum \limits_{k = 1}^{{p_{o} }} y_{{k_{i} }} x_{i}$$

where $n$ is the total number of clones under evaluation; $p_{o}$ is the number of traits considered for maximization and, therefore, subjected to constraints; $y_{{k_{i} }}$ is the value of the EBLUP of genotypic effect divided by the overall mean of the trait $k$, for genotype $i$; $x_{i}$ is a binary variable with value $0$ if genotype $i$ is not selected and $1$ if genotype $i$ is selected;

*subject to the constraints*

$$\mathop \sum \limits_{i = 1}^{n} x_{i} = s$$

where $s$ is the number of clones to be selected (size of the polyclonal group); and, for any given trait k = 1, …, p_c_ (which may, or may not, be included in the objective function):

$$\mathop \sum \limits_{i = 1}^{n} y_{{k_{i} }} x_{i} \ge l_{k},\, for\,k = 1, \ldots ,p_{c}$$

where $p_{c}$ is the number of traits considered for selection constraints; $l_{k}$ is the *right-hand side* value and is defined for trait $k$ as:

$$l_{k} = R_{k} \times s{/}100$$

with $R_{k} \ge 0$ being the minimum desirable genetic gain for trait $k$.

The coefficients of the decision variables in both the IP objective function and the constraints are the genotypic effects EBLUPs (divided by the trait means) which are continuous values, however, the decision variables are binary $x_{i} \in \left\{ {0,1} \right\}$, and therefore, this is an integer programming problem. It is possible to use different traits in the objective function and in the constraints.

The *right-hand side* values of the trait constraints are related to the acceptable minimum values for each trait, that is, the desired minimum gains $\left( {R_{k} } \right)$. The minimum desirable genetic gain indicated for a given trait, which allows to assign different importance to each trait, is directly related to the “economic weight” (higher yield; or higher berry quality traits implying higher wine quality, etc.). Therefore, in the proposed method, the weights for the different traits are defined by constraints and their definition can have several options according to the objectives of viticulture and oenology. When the intention is only to avoid losses in every trait, $R_{k}$ is zero for all these traits. This is called the *base situation*. In addition to the base situations, other problems were also considered. This latter type of situations is referred to in this work as *specific situations* and differs according to the variety. They are described in detail in Table 2, with $R_{k}$ expressed as a percentage of the overall mean for the related trait. In addition, the concepts of maximum possible genetic gain $\left( {R_{{max_{p}}}} \right)$ and maximum admissible genetic gain $\left({R_{{max_{a}}}} \right)$ for a trait were also considered, as indicated in Table 2.

**Table 2:** Definitions of maximum possible genetic gain $\left( {R_{{max_{p} }} } \right)$ and maximum admissible genetic gain $\left( {R_{{max_{a} }} } \right)$ for each trait, as well as some situations that were considered in the application of integer programming based on several traits. See the text for a fuller discussion of the specific situations for each variety

$R_{{max_{p} }}$	The mean of the EBLUPs of the genotypic effects of the best $s$ clones in a given trait, as a percentage of the overall mean
$R_{{max_{a} }}$	The maximum genetic gain in one trait that can be achieved without decreasing any of the other traits, as a percentage of the overall mean
Base situation	Situation where the objective function defined by all traits is maximized and the desired minimum gain for each trait is 0, i.e., $R_{k} = 0{\text{\%}}$, $\forall k$
Specific situations	Encruzado—the objective function based on all traits is maximized, and the desired minimum gain for each trait is: $R_{PA} = 2{\text{\%}}$, $R_{TA} = 2{\text{\%}},R_{YD} = 0\% ,R_{pH} = 0{\text{\%}}$, $R_{BW} = 0{\text{\%}}$; Gouveio—the objective function based on all traits is maximized, and the desired minimum gain for each trait is: $R_{YD} = 20{\text{\%}}$, $R_{PA} = 3{\text{\%}},$ $R_{TA} = 3{\text{\%}}$, $R_{pH} = 1{\text{\%}}$, $R_{BW} = 2{\text{\%}}$; Grenache—the maximum admissible gain for each trait when the desired minimum gain for AC is: $R_{AC} = 15{\text{\%}}$; Rufete—the objective function based only on traits PA and pH is maximized, and the desired minimum gain for each trait is: situation 1) $R_{YD} = 0{\text{\%}}$, $R_{PA} = 0{\text{\%}}$, $R_{pH} = 0{\text{\%}}$, $R_{BW} = 0{\text{\%}}$; situation 2) $R_{YD} = 10{\text{\%}}$, $R_{PA} = 0{\text{\%}}$, $R_{pH} = 0{\text{\%}}$, $R_{BW} = 0{\text{\%}}$; situation 3) $R_{YD} = 10{\text{\%}}$, $R_{PA} = 0{\text{\%}}$, $R_{pH} = 1{\text{\%}}$, $R_{BW} = 0{\text{\%}}$

*YD* yield; *PA* potential alcohol; *TA* total acidity; *BW* berry weight; *AC* anthocyanin content; *TP* total phenols index

To determine both $R_{{max_{p} }}$ and $R_{{max_{a} }}$, for trait $k$, the IP problem is reduced to the maximization of the objective function:

$$z = \mathop \sum \limits_{i = 1}^{n} y_{{k_{i} }} x_{i}$$

but in the case of $R_{{max_{p} }}$ the objective function is subjected to the constraint $\sum\nolimits_{{i = 1}}^{n} {x_{i} = s}$, while in the case of $R_{{max_{a} }}$ the constraints $\sum\nolimits_{{i = 1}}^{n} {y_{{k_{i} }} x_{i} \ge 0}$ are considered for all traits of interest.

#### Correlation between traits

Correlations between traits were calculated between the EBLUPs of the genotypic effects of all possible pairs of traits. The purpose of this analysis was to provide a foundation for the discussion of the genetic gain results for the different polyclonal selections that were tested. In particular, it was intended to support the discussion about the performance of the IP method in cases where there are correlations between traits that counter the selection criteria. The null hypothesis that the correlation between two traits is zero was tested (significance level 0.05). If significant, the correlations were classified according to their absolute values as very weak, weak, moderate, and strong for values below 0.40, between 0.40 and 0.60, between 0.60 and 0.80, and above 0.80, respectively.

### Software

**Fig. 1:**
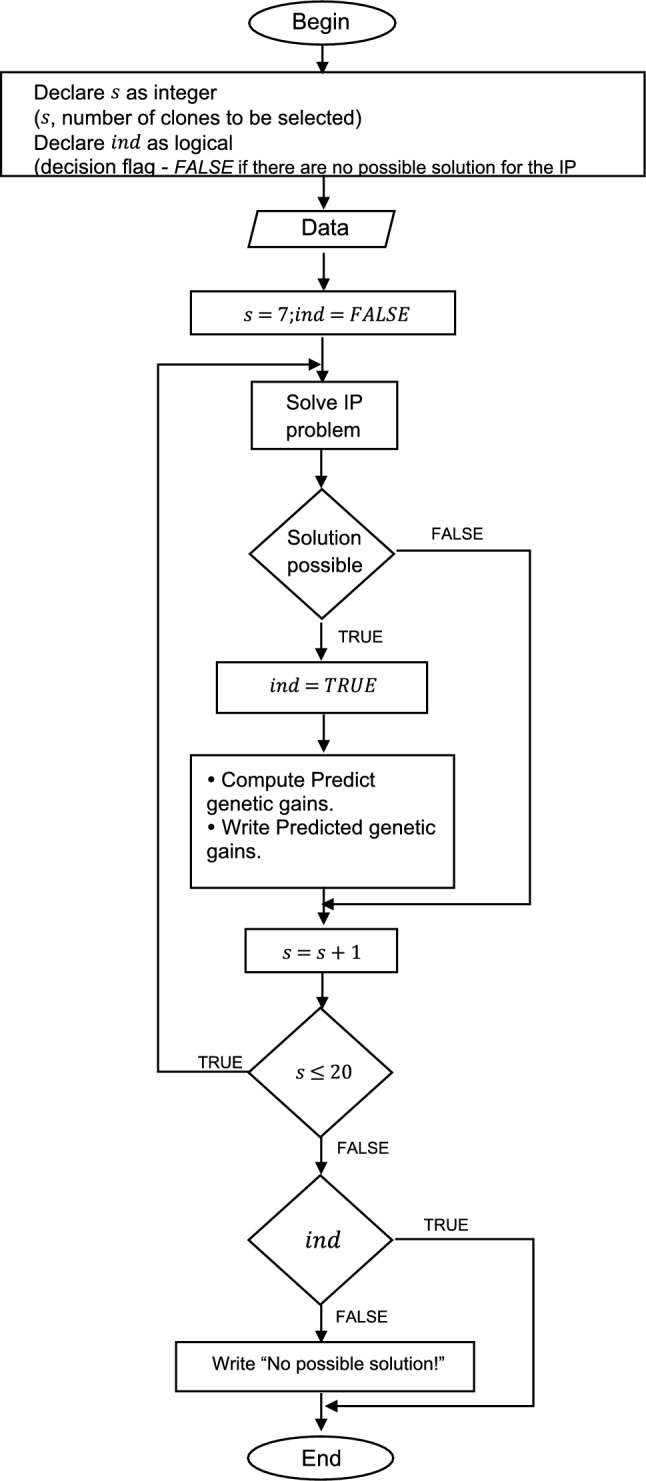
Algorithm used to optimize the application of integer programming (IP) in the polyclonal selection of ancient grapevine varieties

Mixed models were fitted with the package ASReml-R (Butler et al. 2017) for software R (R Core Team 2022). IP problems were solved with software R by a function developed for the effect. This function relies on the R package lpSolve (Berkelaar et al. 2023) and follows the algorithm described in Fig. 1.

## Results

For all the varieties studied, the overall mean, the genotypic variance component estimate, the genotypic coefficient of variation, the broad-sense heritability, and the range of EBLUPs of genotypic effects for all the evaluated traits are shown in Table 3. In terms of within-variety variability, significant genotypic variability was found at the 0.05 significance level for all traits in all varieties, except for TP in variety Rufete. For all varieties, YD was the trait with higher intravarietal variability, while pH was the trait with the lowest values for the coefficient of genotypic variation. Broad-sense heritability was highest for YD and PA, and only for variety Rufete was lower than 0.30 for TA, AC, and TP. For this reason, these three traits were not used for the exercise of polyclonal selection performed in this variety. For all varieties, the range of the EBLUPs of the genotypic effects was largest for YD and smallest for pH.

**Table 3:** For each variety and trait, the overall mean, the estimate of the genotypic variance component $\left( {\hat{\sigma }_{g}^{2} } \right)$ and its standard error (SE), the coefficient of genotypic variation (CV_G_ %), the broad-sense heritability (H^2^), and the minimum and maximum empirical best linear unbiased predictor (EBLUP) of genotypic effect as a percentage of the overall mean and respective range

Variety	Trait	Overall mean	$\hat{\sigma }_{g}^{2}$(SE)*	CV_G_ (%)	H^2^	EBLUP of genotypic effect (% overall mean)		
						Minimum	Maximum	Range
Encruzado	YD	2.921	0.273 (0.042)	17.89	0.695	− 41.70	30.23	71.93
	PA	11.385	0.173 (0.028)	4.48	0.672	− 8.39	9.34	17.73
	TA	7.785	0.178 (0.040)	3.71	0.500	− 13.83	11.16	24.99
	pH	3.414	0.001 (0.000)	0.78	0.463	− 1.44	1.44	2.88
Gouveio	YD	3.517	0.692 (0.096)	23.65	0.836	− 42.82	56.90	99.72
	PA	12.760	0.321 (0.043)	4.44	0.865	− 13.47	8.44	21.91
	TA	4.495	0.088 (0.018)	6.60	0.593	− 10.28	19.09	29.37
	pH	3.927	0.006 (0.001)	1.97	0.845	− 3.97	3.72	7.69
	BW	1.653	0.012 (0.002)	6.63	0.713	− 14.16	14.82	28.98
Grenache	YD	5.213	1.165 (0.184)	20.71	0.808	− 56.88	39.06	95.94
	PA	13.979	0.650 (0.111)	5.77	0.759	− 17.15	6.87	24.02
	TA	3.300	0.086 (0.015)	8.89	0.728	− 14.79	30.15	44.94
	pH	3.817	0.008 (0.002)	2.34	0.715	− 5.74	3.56	9.30
	BW	1.712	0.010 (0.002)	5.84	0.624	− 23.36	11.39	34.75
	AC	179.091	1222.12 (258.38)	19.52	0.623	− 42.88	42.15	85.03
	TP	458.400	1981.33 (896.35)	9.71	0.316	− 13.00	21.96	34.96
Rufete	YD	3.840	0.274 (0.036)	13.63	0.713	− 41.07	28.70	69.77
	PA	13.470	0.163 (0.026)	3.00	0.601	− 8.46	6.17	14.63
	TA	3.282	0.021 (0.007)	4.41	0.296	− 5.61	13.07	18.68
	pH	4.109	0.002 (0.000)	1.09	0.603	− 2.24	1.90	4.14
	BW	2.449	0.039 (0.006)	8.06	0.655	− 18.17	15.39	33.56
	AC	252.900	1034.71 (376.09)	12.72	0.288	− 18.24	25.03	43.27
	TP	571.600	1189.70 (1105.26)	6.03	0.117	− 5.54	8.58	14.12

*YD* yield (kg/plant); *PA* potential alcohol (%v/v); *TA* total acidity (g of tartaric acid/l); *BW* berry weight (g/berry); *AC* anthocyanin content (mg/l); *TP* total phenols index

*The residual likelihood ratio test for the genotypic variance component was performed ($H_{0} :\sigma_{g}^{2} = 0 vs H_{1} :\sigma_{g}^{2} > 0)$. For all studied cases, p-value < 0.05, except in the case of TP in the variety Rufete

Considering the correlations of EBLUPs of genotypic effects between traits, 32% were not significant. Among the significant correlations, 45% were very weak, 29% weak, 16% moderate, and 6% strong (Table 4). YD and BW showed either non-significant, weak, or very weak correlations with all other traits, for every variety. For the variety Encruzado there was only a negative moderate correlation between TA and PH, while for the varieties Gouveio and Rufete all significant correlations were either weak or very weak. Grenache was the variety with the strongest correlations between traits. There were two positive strong correlations between AC and both PA and TP. There were also four moderately strong correlations: two of them positive, between PA and both pH and TP, and two of them negative, between TA and both PA and AC.

**Table 4:** Correlations of the empirical best linear unbiased predictors (EBLUPs) of the genotypic effects between traits for the varieties studied

Variety	Correlations of the EBLUP of genotypic effects						
Encruzado		PA	TA	PH			
	YD	− **0.46**	0.05	− **0.23**			
	PA		**-0.48**	**0.51**			
	TA			− **0.61**			
Gouveio		PA	TA	PH	BW		
	YD	− 0.04	0.03	− **0.43**	− 0.09		
	PA		− **0.18**	**0.50**	**0.26**		
	TA			− **0.52**	0.03		
	PH				0.16		
Grenache		PA	TA	pH	BW	AC	TP
	YD	**0.33**	− **0.30**	**0.29**	0.05	**0.23**	**0.22**
	PA		− **0.74**	**0.73**	**0.18**	**0.83**	**0.67**
	TA			− **0.55**	− 0.02	− **0.69**	− **0.56**
	pH				**0.28**	**0.44**	**0.32**
	BW					− 0.02	− 0.03
	AC						**0.84**
Rufete		PA	PH	BW			
	YD	− 0.09	− **0.43**	0.09			
	PA		**0.30**	**0.25**			
	PH			**0.27**			

*YD* yield; *PA* potential alcohol; *TA* total acidity; *BW* berry weight; *AC* anthocyanin content; *TP* total phenols index

Numbers in bold refer to significant correlations using a significance level of 0.05

The predicted genetic gains obtained with polyclonal selection for the different situations tested in this study are presented in Tables 5, 6, 7, and 8 for the varieties Encruzado, Gouveio, Grenache, and Rufete, respectively. As expected, the maximum admissible gain (${R}_{{max}_{a}}$) was usually smaller than the maximum possible gain (${R}_{{max}_{p}}$), but in most cases not substantially so. For Encruzado variety, both ${R}_{{max}_{p}}$ and ${R}_{{max}_{a}}$ were close to zero for pH, while Grenache showed the largest difference between ${R}_{{max}_{p}}$ and ${R}_{{max}_{a}}$ with values of ${R}_{{max}_{a}}$ for TA and pH about half those of ${R}_{{max}_{p}}$. In Rufete, ${R}_{{max}_{p}}$ was equal (to one decimal place) to ${R}_{{max}_{a}}$ for YD for all sizes of the selected group.

**Table 5:** Values obtained in the variety Encruzado for the predicted maximum possible genetic gains $\left( {Rmax_{p} } \right)$, maximum admissible genetic gains $\left( {Rmax_{a} } \right)$, genetic gains considering the base situation, and the specific situations defined in Table 2

	Trait	Size of the polyclonal group (from 7 to 20 clones)													
		7	8	9	10	11	12	13	14	15	16	17	18	19	20
$Rmax_{p}$	YD	26.6	26.3	26.0	25.7	25.4	25.1	24.9	24.6	24.3	24.0	23.7	23.5	23.2	23.0
	PA	7.0	6.8	6.6	6.5	6.3	6.2	6.1	5.9	5.8	5.7	5.6	5.5	5.4	5.3
	TA	8.5	8.3	8.1	7.9	7.7	7.5	7.4	7.2	7.1	7.0	6.9	6.8	6.7	6.6
	pH	0.5	0.5	0.5	0.5	0.5	0.5	0.4	0.4	0.4	0.4	0.4	0.4	0.4	0.4
$Rmax_{a}$	YD	25.6	25.5	25.0	24.7	24.4	24.1	23.8	23.6	23.3	23.0	22.7	22.6	22.1	22.0
	PA	3.9	4.0	3.9	3.8	3.8	3.8	3.7	3.7	3.7	3.6	3.6	3.6	3.5	3.5
	TA	6.7	6.5	6.2	6.1	6.0	6.0	5.9	5.8	5.7	5.6	5.5	5.5	5.4	5.3
	pH	0.4	0.4	0.4	0.4	0.4	0.4	0.4	0.3	0.3	0.3	0.3	0.3	0.3	0.3
Base situation	YD	25.5	25.5	25.0	24.5	24.2	23.6	23.0	22.9	22.8	22.7	22.4	22.4	22.1	21.6
	PA	0.1	0.0	0.1	0.0	0.1	0.0	0.0	0.0	0.0	0.0	0.1	0.0	0.0	0.0
	TA	0.9	1.0	0.7	1.2	0.9	1.5	1.5	1.2	1.1	0.8	0.7	0.5	0.4	0.5
	pH	0.0	0.0	0.0	0.0	0.0	0.0	0.0	0.0	0.0	0.0	0.0	0.0	0.0	0.0
Specific situation	YD	10.4	9.5	9.3	9.2	9.1	8.2	8.0	7.4	7.0	6.8	6.4	6.3	5.9	5.3
	PA	2.1	2.0	2.1	2.0	2.0	2.0	2.0	2.0	2.0	2.0	2.0	2.0	2.0	2.0
	TA	2.0	2.2	2.1	2.0	2.0	2.1	2.0	2.0	2.1	2.0	2.0	2.0	2.0	2.0
	pH	0.0	0.1	0.1	0.0	0.1	0.1	0.0	0.0	0.0	0.1	0.0	0.0	0.0	0.0

*YD* yield (kg/plant); *PA* potential alcohol (%v/v); *TA* total acidity (g of tartaric acid/l); *BW* berry weight (g/berry); *AC *anthocyanin content (mg/l); *TP* total phenols index

**Table 6:** Values obtained in the variety Gouveio for the predicted maximum possible genetic gains $\left( {Rmax_{p} } \right)$, maximum admissible genetic gains $\left( {Rmax_{a} } \right)$, genetic gains considering the base situation, and the specific situations defined in Table 2

	Trait	Size of the polyclonal group (from 7 to 20 clones)													
		7	8	9	10	11	12	13	14	15	16	17	18	19	20
$Rmax_{p}$	YD	49.4	48.4	47.4	46.6	46.0	45.3	44.6	43.9	43.1	42.4	41.6	40.9	40.2	39.5
	PA	6.7	6.6	6.6	6.5	6.4	6.4	6.3	6.3	6.2	6.1	6.1	6.0	6.0	5.9
	TA	12.5	12.2	11.8	11.3	11.0	10.6	10.4	10.1	9.8	9.6	9.3	9.1	8.9	8.7
	pH	3.5	3.4	3.3	3.2	3.2	3.1	3.1	3.1	3.0	3.0	2.9	2.9	2.9	2.8
	BW	11.8	11.4	11.1	10.8	10.6	10.3	10.1	9.9	9.7	9.6	9.4	9.3	9.1	9.0
$Rmax_{a}$	YD	42.7	42.3	41.6	41.4	40.7	40.3	40.0	39.7	39.3	39.0	38.8	38.2	37.9	37.7
	PA	6.0	5.9	5.8	5.6	5.5	5.4	5.4	5.3	5.2	5.2	5.1	5.1	5.0	4.9
	TA	10.9	10.5	10.2	10.0	9.8	9.6	9.5	9.3	9.2	9.0	8.9	8.7	8.7	8.5
	pH	2.7	2.6	2.6	2.5	2.5	2.5	2.4	2.4	2.4	2.4	2.3	2.3	2.3	2.3
	BW	10.8	10.5	10.3	10.1	9.9	9.8	9.6	9.3	9.1	8.9	8.7	8.5	8.4	8.3
Base situation	YD	40.8	39.1	40.2	38.3	39.2	37.9	37.5	38.0	37.3	37.5	37.9	36.7	36.9	37.1
	PA	0.1	0.1	0.0	0.1	0.0	0.1	0.0	0.1	0.0	0.0	0.0	0.0	0.2	0.1
	TA	2.6	3.2	3.1	3.4	3.2	3.1	2.8	2.7	2.7	2.3	2.3	2.6	2.1	2.0
	pH	1.6	1.8	1.9	1.9	1.9	1.8	1.8	1.8	1.8	1.8	1.9	1.8	1.8	1.9
	BW	5.4	5.0	3.8	4.4	3.5	3.9	4.1	3.6	3.9	3.2	2.7	3.3	2.7	2.4
Specific situation	YD	24.2	21.0		21.4	*	*	*	*	*	*	*	*	*	*
	PA	3.1	3.0		3.0										
	TA	3.3	4.0	*	3.3										
	pH	1.0	1.0		1.1										
	BW	2.3	2.1		2.3										

*YD* yield (kg/plant); *PA* potential alcohol (%v/v); *TA* total acidity (g of tartaric acid/l); *BW* berry weight (g/berry); *AC* anthocyanin content (mg/l); *TP* total phenols index

*For the specific situation defined in Table 2, no solutions were found for the polyclonal groups of size 9 and 11 to 20

**Table 7:** Values obtained in the variety Grenache for the predicted maximum possible genetic gains (${\text{Rmax}}_{\text{p}}$), maximum admissible genetic gains (${\text{Rmax}}_{\text{a}}$), genetic gains considering the base situation, and the specific situations defined in Table 2

	Trait	Size of the polyclonal group (from 7 to 20 clones)													
		7	8	9	10	11	12	13	14	15	16	17	18	19	20
${Rmax}_{p}$	YD	30.6	29.8	29.1	28.5	28.0	27.4	26.8	26.3	25.9	25.5	25.1	24.8	24.4	24.1
	PA	6.3	6.2	6.2	6.1	6.1	6.0	5.9	5.9	5.8	5.7	5.7	5.6	5.6	5.5
	TA	18.7	17.9	17.4	16.9	16.5	16.1	15.6	15.1	14.6	14.2	13.8	13.5	13.2	12.8
	pH	4.6	4.5	4.4	4.4	4.3	4.2	4.1	4.0	3.9	3.8	3.7	3.6	3.6	3.5
	BW	11.2	10.5	10.0	9.6	9.2	8.9	8.6	8.4	8.1	7.9	7.7	7.5	7.4	7.2
	AC	31.6	30.5	29.6	28.8	28.0	27.2	26.5	25.9	25.3	24.7	24.2	23.6	23.2	22.8
	TP	11.7	11.2	10.8	10.6	10.3	10.0	9.7	9.5	9.3	9.1	8.9	8.8	8.6	8.4
${Rmax}_{a}$	YD	29.4	28.7	27.7	26.9	26.4	25.9	25.7	25.1	24.7	24.1	23.6	23.4	22.9	22.6
	PA	4.2	4.3	4.1	4.0	4.0	3.9	3.9	3.8	3.7	3.7	3.6	3.6	3.6	3.5
	TA	9.0	9.0	9.1	8.7	8.6	8.3	8.2	8.0	7.9	7.8	7.6	7.5	7.3	7.2
	pH	2.7	2.7	2.6	2.6	2.5	2.4	2.4	2.3	2.3	2.2	2.2	2.2	2.1	2.1
	BW	10.1	9.7	9.3	9.0	8.7	8.2	8.0	7.8	7.5	7.2	7.0	6.9	6.7	6.5
	AC	22.7	21.9	21.3	21.0	20.5	20.2	19.8	19.2	18.8	18.8	18.3	17.8	17.6	17.4
	TP	10.3	9.9	9.7	9.4	8.9	8.6	8.4	8.2	7.9	7.7	7.5	7.4	7.3	7.1
Base situation	YD	15.8	19.1	14.9	17.3	15.8	16.4	17.7	17.5	17.7	13.6	13.7	14.0	14.2	14.1
	PA	3.4	3.0	3.4	3.1	3.3	2.9	2.6	2.7	2.4	2.7	2.5	2.5	2.3	2.4
	TA	0.3	0.2	0.2	0.3	0.1	0.1	0.1	0.1	0.0	0.1	0.1	0.0	0.1	0.1
	pH	0.3	0.3	0.1	0.0	0.1	0.1	0.1	0.1	0.2	0.0	0.1	0.0	0.1	0.0
	BW	5.0	1.0	4.1	1.2	2.7	2.5	1.9	2.3	1.9	1.6	1.8	2.2	1.8	1.5
	AC	16.3	16.6	16.8	16.8	15.3	14.8	13.1	13.0	11.9	14.6	13.9	13.5	12.9	12.5
	TP	6.8	6.6	6.4	6.1	7.1	5.8	6.4	5.0	5.5	6.4	6.1	5.4	5.4	5.6
Specific situation	YD	20.4	19.1	18.9	18.6	18.1	17.3	16.2	15.5	15.4	14.3	12.7	12.4	11.4	10.5
	PA	4.1	4.2	4.1	4.0	3.9	3.9	3.8	3.8	3.7	3.7	3.6	3.5	3.5	3.5
	TA	4.0	3.7	3.7	3.3	3.3	2.8	2.9	2.6	2.3	2.2	2.0	1.8	1.6	1.5
	pH	1.9	1.9	1.8	1.7	1.6	1.5	1.4	1.3	1.3	1.2	1.1	1.1	1.0	0.9
	BW	7.2	7.2	6.4	6.4	6.2	5.9	5.7	5.4	5.2	4.8	4.8	4.4	4.1	4.0
	AC	> = 15	> = 15	> = 15	> = 15	> = 15	> = 15	> = 15	> = 15	> = 15	> = 15	> = 15	> = 15	> = 15	> = 15
	TP	10.3	9.9	9.7	9.4	8.9	8.6	8.4	8.2	7.9	7.7	7.5	7.3	7.2	7.0

*YD* yield (kg/plant); *PA* potential alcohol (%v/v); *TA* total acidity (g of tartaric acid/l); *BW* berry weight (g/berry); *AC *anthocyanin content (mg/l); *TP* total phenols index

**Table 8:** Values obtained in the variety Rufete for the predicted maximum possible genetic gains (${Rmax}_{p}$), maximum admissible genetic gains (${Rmax}_{a}$), genetic gains considering the base situation, and the specific situations defined in Table 2

	Trait	Size of the polyclonal group (from 7 to 20 clones)													
		7	8	9	10	11	12	13	14	15	16	17	18	19	20
${Rmax}_{p}$	YD	23.3	22.7	22.3	21.9	21.5	21.2	20.9	20.5	20.2	20.0	19.7	19.5	19.3	19.0
	PA	4.3	4.2	4.2	4.1	4.1	4.0	4.0	3.9	3.9	3.8	3.8	3.8	3.7	3.7
	pH	1.8	1.7	1.7	1.6	1.6	1.6	1.5	1.5	1.5	1.5	1.4	1.4	1.4	1.4
	BW	15.5	15.3	15.2	15.0	14.7	14.5	14.3	14.1	13.9	13.7	13.5	13.3	13.1	13.0
${Rmax}_{a}$	YD	23.3	22.7	22.3	21.9	21.5	21.2	20.9	20.5	20.2	19.9	19.7	19.5	19.3	19.0
	PA	3.8	3.7	3.7	3.7	3.6	3.6	3.5	3.5	3.5	3.5	3.4	3.4	3.4	3.4
	pH	1.5	1.5	1.4	1.4	1.4	1.4	1.4	1.4	1.3	1.3	1.3	1.3	1.3	1.3
	BW	14.5	14.3	14.3	14.1	13.7	13.3	13.1	12.7	12.5	12.2	12.0	11.8	11.7	11.5
Base situation	YD	20.3	20.2	19.9	19.8	19.4	18.7	18.7	18.6	18.4	17.6	17.5	17.3	16.8	16.6
	PA	0.0	0.3	0.7	0.3	0.3	0.1	0.2	0.0	0.2	0.1	0.1	0.1	0.1	0.2
	pH	0.2	0.4	0.2	0.1	0.1	0.3	0.3	0.4	0.3	0.3	0.3	0.3	0.4	0.3
	BW	6.8	5.9	5.4	5.7	5.6	5.9	5.3	5.1	4.8	5.4	5.2	5.0	5.2	5.1
Specific															
Situation 1	YD	0.2	0.6	1.0	0.2	0.9	0.1	0.8	0.8	1.4	0.6	0.0	0.3	0.1	0.3
	PA	3.6	3.6	3.6	3.5	3.4	3.4	3.4	3.3	3.3	3.3	3.3	3.3	3.3	3.4
	pH	0.3	0.2	0.1	0.2	0.2	0.3	0.2	0.2	0.2	0.1	0.1	0.1	0.1	0.0
	BW	1.5	1.9	1.6	1.6	1.3	1.6	1.2	1.0	0.5	0.5	0.6	0.1	0.1	0.1
Situation 2	YD	10.0	10.0	10.1	10.0	10.4	10.0	10.0	10.1	10.1	10.1	10.0	10.0	10.1	10.0
	PA	3.3	3.4	3.2	3.3	3.2	3.1	3.1	3.1	3.1	2.9	2.9	2.9	2.8	2.8
	pH	0.1	0.0	0.1	0.0	0.1	0.1	0.1	0.0	0.0	0.1	0.2	0.1	0.1	0.2
	BW	0.8	0.7	0.7	0.3	0.3	0.4	0.0	0.2	0.1	0.0	0.3	0.3	0.1	0.1
Situation 3	YD	10.2	10.5	10.2	10.1	10.0	10.1	10.1	10.5	10.1	10.3	10.2	10.0	10.0	10.0
	PA	1.6	1.5	1.4	1.4	1.3	1.3	1.2	1.1	1.1	1.0	1.0	0.9	0.8	0.8
	pH	1.0	1.0	1.0	1.0	1.0	1.0	1.0	1.0	1.0	1.0	1.0	1.0	1.0	1.0
	BW	1.2	1.0	1.1	0.4	0.6	0.5	1.1	0.5	0.2	0.4	0.6	1.0	0.8	0.9

*YD* yield (kg/plant); *PA* potential alcohol (%v/v); *TA* total acidity (g of tartaric acid/l); *BW* berry weight (g/berry); *AC* anthocyanin content (mg/l); *TP* total phenols index

For all varieties, it was possible to find a solution in the base situation, selecting a group of clones for all the specified cardinalities without compromising any trait. This was the case even for Grenache (Table 7), which was the variety with most traits considered in the selection. However, for some traits the gains obtained were zero or very small, as was the case, for all varieties, in PA, TA, and pH. For both Encruzado and Gouveio varieties (Tables 5 and 6, respectively), gains close to ${R}_{{max}_{a}}$ were obtained for YD, but in Encruzado no gains were obtained for the other traits.

For the specific situations described in Table 2, the results depended on the variety. For the variety Encruzado (Table 5), it was possible to obtain a group of every desired size for the specific situation described in Table 2. The constraints of a minimum desirable gain of 2% for PA and TA allowed a gain for YD that ranged from 10.4% to 5.3% for the groups of 7 clones and 20 clones, respectively. For the specific situation defined for variety Gouveio (Table 6), solutions were found only for groups of 7, 8, and 10 clones. In fact, for the solutions found, gains in YD were always greater than 20% and all the other traits respected the constraints established in Table 2. For the specific situation defined for Grenache (Table 7), it was possible to obtain a group of every desired size. The results showed that, considering the desirable minimum gain of 15% or more for AC, the new maximum admissible gains obtained for TA and pH were almost half of those obtained for ${R}_{{max}_{a}}$, and for PA and TP they were similar to those obtained for ${R}_{{max}_{a}}$.

For the Rufete variety (Table 8), three specific situations are defined in Table 2 to try to find more balanced gains than those obtained in the base situation, where approximately zero gains were obtained for PA and pH. To overcome this result, it was decided to include only PA and pH in the objective function. In the first specific situation defined in Table 2 for Rufete, for all the traits analyzed, the desired minimum gains were equal to or greater than zero. The results showed that it was possible to obtain a solution for all groups of each size with a gain close to ${R}_{{max}_{a}}$ for PA, but for YD and BW the gains obtained were much lower than those obtained for ${R}_{{max}_{a}}$ and in the base situation. Given the high importance of yield (YD), in the second specific situation defined in Table 2 for Rufete, the constraint of a minimum desired gain of 10% for YD was considered. The results showed that it was possible to obtain a solution for all groups of each size, although the gains of pH and BW were close to zero. Finally, in the third specific situation defined in Table 2 for Rufete, the constraints of considering a minimum desired gain of 10% for YD and 1% for pH were considered. The results showed that it was possible to obtain a solution for all groups of each size, obtaining more balanced gains between PA, pH and BW, and still obtaining a 10% gain in YD.

## Discussion

Integer programming (IP) was used as a novel approach for selection within ancient grapevine varieties. Several selection criteria were considered using real data to test the method, which was successfully applied to perform polyclonal selection. The cases presented and studied are only examples to illustrate the application and versatility of this method. Other varieties, traits, and selection criteria could have been established. The methodology under discussion offers a novelty in the form of the possibility to define different selected groups with minimum desirable genetic gains for the traits in question, allowing to give different importance (equivalent to “economic weights”) to each trait in the constraints. This added value of the methodology is evident in its ability to allow the selection of the group that better meets the defined objectives and directly responds to the needs of the vine and wine sector.

As expected, the higher the genetic intra-varietal variability the greater the possibility of obtaining greater genetic gains. In fact, the Gouveio and Grenache varieties that showed the highest intravarietal trait variability were also the varieties that obtained higher genetic gains for the different options of the selection criteria established in Table 2. This reinforces the importance of a high intravarietal genetic variability for the success of the selection program.

In addition to the good results for polyclonal selection considering several target traits, the versatility of this method, namely, providing the possibility of predicting the maximum admissible genetic gain for each trait, is very helpful in defining the desired minimum gains. This information is not available with other methods and is very useful when selecting for several traits simultaneously. The classical selection methods referred to for multiple trait selection are the Tandem, Independent Culling Levels, and Total Score (Selection Index) methods (Hazel and Lush 1942), which are mainly based on phenotypic values. In tandem selection, selection is made one trait at a time over several generations (Cotterill and Dean 1990), so that selection on a trait begins when the desired values for the previous trait are achieved, and therefore selection for multiple traits is made successively rather than simultaneously. Index selection allows the simultaneous selection of several traits and its efficiency lies in the fact that, by definition, a high gain in one trait of a given genotype can compensate for its decrease in another trait (Cotterill and Dean 1990), since in most indices the traits are weighted by economic value. Some indices with restrictions have been proposed to avoid losses (Kempthorne and Nordskog 1959) or to specify minimum desired gains (Pesek and Baker 1969), but in these cases the objective is achieved by the economic weights. On the other hand, independent culling levels selection can be carried out simultaneously for the different traits; however, the main difficulty lies in choosing the appropriate culling levels (Cotterill and Dean 1990). Bos and Caligari (2008) presented some suggestions for setting culling levels but mentioned that these have not yet been studied.

The ability to calculate the maximum admissible gain for each trait appears to be one an advantage of the method proposed over existing methods, as it allows to define realistic minimum desired gains. Not only can the desired minimum gain (${R}_{k}$) be used to avoid losses or to set a minimum increase in a trait, but it is also possible to set a cap on the increase, thus defining an acceptable interval. With this method it is possible to obtain different groups for each cardinality under the same conditions. If for some reason one does not want to select a clone in a group chosen by the IP solution, it can be forced not to be selected by adding a new constraint that specifies that the variable that refers to the clone is equal to 0. When solving the new IP problem, a new group of clones is accessed for the same size, if the problem remains possible. Furthermore, this group can differ from the first not only in the excluded clone, since other clones can be substituted, but also in the value of the new objective function. Therefore, an important difference between this method and the selection indices mentioned above is that the selection indices are designed to identify individual genotypes that meet a specified criterion, whereas IP facilitates the selection of a group that fulfills a particular condition which is exactly the rationale behind polyclonal selection, thus justifying its application in this type of selection. In this work, it was possible to obtain a group of each desired size for the base situation of all varieties, even in Grenache with the selection based on seven traits. According to the algorithm used in this work, all group sizes are tested, and only feasible results are reported. The specific situation for Gouveio is an example of this, since for the imposed desired minimum gains (${R}_{k}$) only groups of 7, 8, and 10 clones were found, which means that the constraints must be changed if other group cardinalities are desired.

Theoretically, it is possible that there are some varieties for which, even in the base situation, the conditions cannot be met for some group size, i.e., the IP problem has no feasible solution. From a selection point of view, this means that it is impossible to select groups of the desired dimension without losses in at least one of the traits considered. There may be several reasons for this, e.g., low intra-varietal variability or strong negative correlations between the target traits considered for selection. However, since the mean of the EBLUPs of the genotypic effects is zero and groups of clones (not individual clones) are selected, the base situation usually has a feasible solution, since a decrease in a trait present in one selected clone can be compensated by an increase for the same trait in another selected clone.

The correlations of the EBLUPs of the genotypic effects between the evaluated traits have been calculated and analyzed because of their high importance in the selection process. If strong positive correlations between traits are observed, indirect selection could be used, allowing a difficult trait to be selected on the basis of another trait that is easier to evaluate. If a strong negative correlation is observed between two traits that are to be maximized, no selection gain can be obtained for either trait, even if they have high heritability (Falconer and Mackay 1996). The negative correlations between TA and pH are expected because the higher the acidity, the lower the pH, but this correlation favors the intention of the selection program to increase TA and decrease pH. On the other hand, the ripening process is responsible for the negative correlation between PA and TA, which was detected, to different degrees, in many varieties. This correlation was moderate in Grenache, weak in Encruzado, and very weak in Gouveio, which is in line with the results obtained by Gonçalves et al. (2016) for three other grapevine varieties. However, in the case of PA and TA the negative correlation counters the intention of increasing both traits, as does the positive correlation between PA and pH. This may be responsible for the null or very low gains obtained in these three traits in all varieties with the base situation.

The influence of inter-trait correlations in the selection process was reflected in the differences between the maximum possible genetic gain $\left( {R_{{max_{p} }} } \right)$ and maximum admissible genetic gain $\left( {R_{{max_{a} }} } \right)$. The largest difference between the values of $R_{{max_{p} }}$ and $R_{{max_{a} }}$ was observed in Grenache, the variety for which the negative correlations between the traits were higher. In this variety, for TA and pH, which had negative and positive correlations, respectively, with all the other traits, the values obtained for $R_{{max_{a} }}$ were almost half of those obtained for $R_{{max_{p} }}$. Considering the specific situation that defined the minimum desirable genetic gain equal or larger than 15% for AC, again the traits that showed a higher decrease in the new admissible genetic gain with respect to the values obtained for $R_{{max_{a} }}$ were TA and pH. The other traits that presented positive correlations with AC decreased less in the new admissible genetic gains when compared to those obtained for $R_{{max_{a} }}$ values. In the cases of PA and TP, which have strong positive correlations with AC, the new values of the admissible genetic gains obtained for the specific situation were similar to those obtained for $R_{{max_{a} }}$. YD and BW, which had no correlations with all the other traits, showed similar results for $R_{{max_{p} }}$ and $R_{{max_{a} }}$. This was also observed for Gouveio and Rufete varieties, where weak correlations between the different traits were found.

In summary, the maximum possible genetic gain for each trait without considering other traits $\left( {R_{{max_{p} }} } \right)$ can be misleading in the sense that it shows how much a trait can be improved, but nothing about the effect of that improvement on the remaining traits. The maximum admissible gain for a trait without decreasing any of the other traits $\left( {R_{{max_{a} }} } \right)$, which can be known using the IP method proposed in this work, is very useful as it allows to assess the impact of the correlations on the gains and to set realistic targets for the selection in terms of the desirable maximum gains, even if the existing correlations counter the goals of the selection program.

The effect of negative correlations affects not only $R_{{max_{a} }}$ but also the gains obtained in the multitrait selection criteria, as can be seen in the base situation of Grenache, where the predicted gains for TA and pH are close to zero. However, knowing $R_{{max_{a} }}$, it is possible to define a desirable minimum gain for the different traits, allowing a polyclonal selection with more balanced gains for the different traits. This was demonstrated in the specific situation of the variety Encruzado and in the third specific situation of the variety Rufete, where in both cases polyclonal materials with more balanced gains of selection were obtained.

It is important to emphasize that the IP problem presented in this work was not formulated for the selection of individual clones (clonal selection). In fact, optimizing the IP problem may result in the selection of individual clones that have gains in some traits but losses in others, but when used in a group (polyclonal selection) the overall objective of the IP problem (and, consequently, the selection criteria) is achieved. This point also reinforces the advantages of polyclonal selection as a method of obtaining more balanced selected material for multiple target traits.

To conclude, the results showed that integer programming can be successfully applied to multitrait polyclonal selection, as it is a tool to select a group of clones with gains in the desired traits while avoiding losses in the others.

## Data Availability

The data sets used in the current study are available from the corresponding author on reasonable request.
